# Metronidazole overexposure in children and its association with new-onset Crohn’s disease (IBD)

**DOI:** 10.1017/ash.2024.20

**Published:** 2024-02-14

**Authors:** Mudassir Nisar, Hamza Ashraf, Haider Ashfaq

**Affiliations:** 1 Department of Medicine, Services Institute of Medical Sciences, Lahore, Punjab, Pakistan; 2 Department of Medicine, Allama Iqbal Medical College, Lahore, Punjab, Pakistan

Metronidazole is an antibiotic and an antiprotozoal medication, and it is administered independently or in conjunction with other antibiotics to treat infections affecting different body parts, including the reproductive system, gastrointestinal tract, nervous system, and more, with some major observed symptoms of diarrhea and vaginal or urethral discharge. Its primary mode of action is to inhibit bacterial growth. The direct link between its mechanism of action and its association with new-onset Crohn’s disease (CD) is an area of active research. It might result from the complex interplay of genetic and environmental factors and the gut microbiome. In a cross-sectional study conducted in Pakistan, it was found that metronidazole ranked as the second most frequently self-administered antibiotic, with a utilization rate of 35.20%.^1^ This highlights the extensive over-the-counter use of this antibiotic for the treatment of various infections and is primarily attributed to a lack of proper diagnosis and reliance on self-prescription instead of seeking guidance from healthcare professionals and laboratories.

According to a meta-analysis, the use of antibiotics in children, specifically metronidazole, was associated with an increased risk of inflammatory bowel disease (IBD), particularly CD (OR: 5.01; 95% CI: 1.65–15.25).^2^ This risk was more pronounced when the antibiotic was taken during the 24 months preceding the index date. The correlation between CD and the use of antibiotics was more pronounced in boys than in girls (*P* = .01).^3^ Also, in a case–control analysis aimed at investigating the potential link between antibiotic usage 2–5 years prior to diagnosis and the development of IBD, it was observed that a somewhat stronger association was present, especially in cases of CD with ≥1 and ≥2 instances of antibiotic dispensation.^4^Another nation-wide study in Denmark from 1995 to 2018 showed that exposure to oral antibiotics within the initial 5 years of life was linked to an elevated risk of developing pediatric inflammatory bowel disease (PIBD) (hazard ratio [HR] = 1.33 with a 95% confidence interval [CI] of 1.2–1.5, *P* < .0001). Additionally, the risk was heightened when patients had received four or more antibiotic prescriptions as opposed to having no antibiotics (HR = 1.33 with a 95% CI of 1.2–1.5, *P* < .0001).^5^ These figures and associations clearly depict that there is an increased risk of CD in infants and young children who are overexposed to antibiotics, specifically metronidazole, which is the most commonly used medication for diarrhea and dysentery.

Based on these investigations, it can be clearly concluded that new-onset CD due to overexposure to metronidazole in children is a critical aspect of pediatric healthcare. Hence, it is important for both medical practitioners and the general public to be aware of the potential harmful side effects of metronidazole usage, especially in children. Healthcare professionals should play a vital role in educating patients, offering counseling, and ensuring the correct use of this pharmaceutical. The absence of strict laws regulating the over-the-counter availability of antibiotics and the limited availability of health insurance further compound this issue. Self-medication and poor diagnostic approach among general public are the major underlying causes. Therefore, it is necessary for the public to be aware of the harmful aspects of self-medication, and they should be aware of the dangers of metronidazole overuse. Lastly, it is advisable to establish legislation for the management of antimicrobial prescriptions.^1^
